# Understanding the Intrinsic Water Wettability of Hexagonal Boron Nitride

**DOI:** 10.1021/acs.langmuir.3c04035

**Published:** 2024-03-14

**Authors:** Fan Yang, Alex D. McQuain, Anumita Kumari, Dhruthi Gundurao, Haitao Liu, Lei Li

**Affiliations:** † Department of Chemical & Petroleum Engineering, Swanson School of Engineering, University of Pittsburgh, Pittsburgh, Pennsylvania 15261, United States; ‡ Department of Chemistry, University of Pittsburgh, Pittsburgh, Pennsylvania 15260, United States

## Abstract

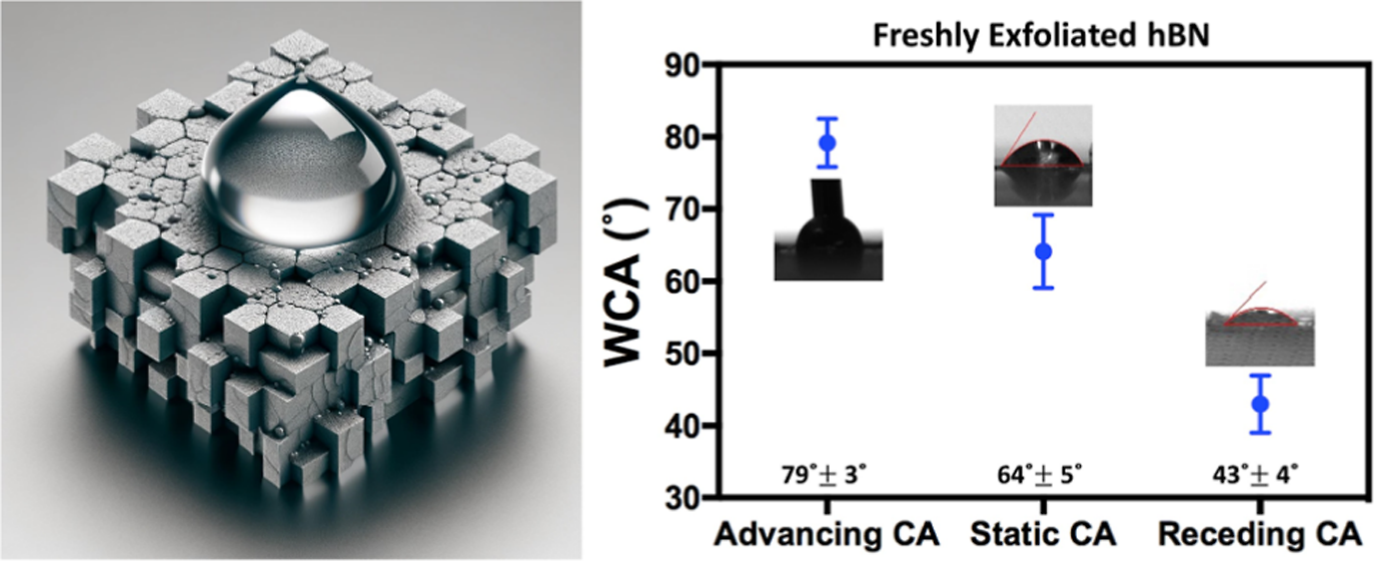

The water wettability of hexagonal boron nitride (hBN) has attracted a lot of research interest in the past 15 years. Experimentally, the static water contact angle (WCA) has been widely utilized to characterize the intrinsic water wettability of hBN. In the current study, we have investigated the effect of airborne hydrocarbons and defects on both static and dynamic WCAs of hBN. Our results showed that the static WCA is impacted by defects, which suggests that previously reported static WCAs do not characterize the intrinsic water wettability of hBN since the state-of-the-art hBN samples always have relatively high defect density. Instead, we found that the advancing WCA of freshly exfoliated hBN is not affected by the defects and airborne hydrocarbons. As a result, the advancing WCA on freshly exfoliated hBN, determined to be 79 ± 3°, best represents the intrinsic water wettability of hBN. A qualitative model has been proposed to describe the effect of airborne hydrocarbons and defects on the static and dynamic WCA of hBN, which is well supported by the experimental results. The finding here has important implications for the water wettability of 2D materials.

## Introduction

Hexagonal boron nitride (hBN) is a heteropolar two-dimensional (2D) material made up of boron and nitrogen atoms arranged in a hexagonal lattice structure, exhibiting significant charge separation between the boron and nitrogen atoms.^1−3^ It has a layered structure and the layers are held together by strong intralayer covalent bonds and weak interlayer van der Waals (vdW) forces, allowing them to easily slide over each other.^2,4^ In recent decades, graphene has been widely studied as a promising material candidate for various nanodevices.^4^ In contrast to graphene, hBN exhibits insulating properties attributed to its substantial band gap of approximately 5.9 eV.^5^ Consequently, there is a compelling incentive to investigate its characteristics as a dielectric material.^6−8^ Due to its exceptional electrical insulating capabilities, high thermal stability, low friction, optical transparency, and inert chemical nature, hBN is promising as anticorrosive,^9^ high-temperature,^10^ low-friction,^11^ and wear-resistant coatings.^11−13^ It also finds applications in optoelectronic apparatuses and functions as a photocatalyst for water treatment.^6,7,12,14,15^ In these applications, the wettability of hBN, affecting its interaction with water, emerges as a pivotal determinant. For instance, the wettability of hBN exert a significant impact on its efficacy as a coating material.^5,16^ Moreover, water wettability is critical to the bonding of conductive substances to hBN surfaces, thus impacting the overall performance of hBN within electronic devices.^8^

**Figure 1:**
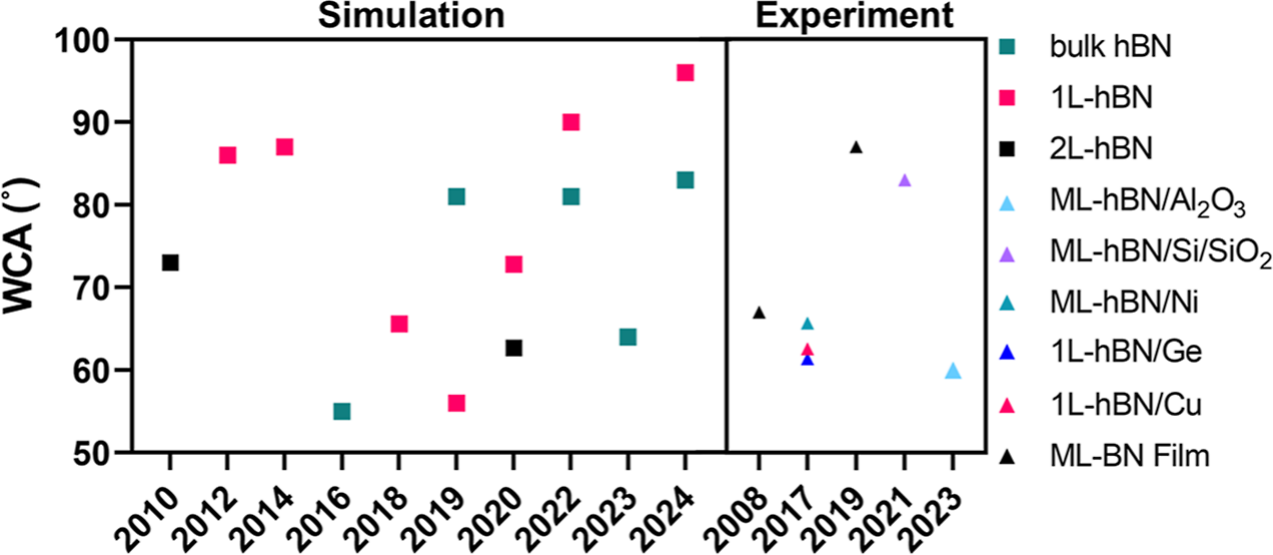
Experimental and theoretical WCA data of hBN. Triangle symbols represent experimental data on monolayer (1L) and multilayer (ML) hBN supported by various substrates: ML-boron nitride (BN) film,^17,18^ 1L-hBN on Cu,^5^ 1L-hBN on Ge,^5^ ML-hBN on Ni,^5^ ML-hBN on Si/SiO_2_,^19^ and ML-hBN on Al_2_O_3_.^20^ Square symbols represent computationally calculated WCA for 1L-hBN, double-layer hBN (2L-hBN), and bulk hBN.^1,16,21−29^

There are significant variations in the reported wettability data for hBN. As depicted in Figure 1, the static water contact angle (WCA) has been reported in prior studies on various hBN samples, including chemical vapor deposition (CVD) grown 1L hBN on Cu/Ge, various ML hBN on Al_2_O_3_, Si/SiO_2_, and bulk boron nitride (BN). Li et al. reported a WCA of 67° for BN films deposited on silicon (100) and quartz substrates utilizing a radio frequency magnetron sputtering system.^17^ In a recent study, Li et al. reported that the WCA on a smooth, extensive CVD-grown hBN on copper, germanium, and nickel surfaces with minimal surface impurities is approximately 61–66°, which is believed to represent the inherent water wettability of the hBN basal plane, unaffected by surface irregularities or hydrocarbon contamination.^5^ Moreover, the observed contact angle suggests that the pristine hBN basal plane exhibits mild hydrophilicity, similar to graphite and molybdenum disulfide (MoS_2_).^30^ Similarly, using an alternative hBN synthesis approach, Biswas et al. grew hBN nanosheets on an aluminum oxide (Al_2_O_3_) substrate at room temperature using a highly energetic pulsed laser deposition (PLD) technique, yielding a WCA of 60°.^20^ However, Keerthi et al. conducted a time-dependent static WCA analysis on mechanically exfoliated bulk hBN, employing 6 M LiCl aqueous solutions and a PicoPump to generate small droplets with an approximate diameter of 100 μm. Notably, the mechanical exfoliation of hBN led to an initial WCA measurement of approximately 83°, which is very different from previously reported results of 60–67° though it is unclear what is the effect of LiCl.^19^ Furthermore, Zhang et al. performed advancing WCA measurements at a controlled low rate on ML BN nanosheets. These films were fabricated by liquid phase exfoliation. An advancing WCA of 87° was reported, which presents a significant deviation from the measured static WCA of 72° for ML BN nanosheets.^18^ Overall, regarding the experimentally measured WCA on hBN, significant controversy exists.

There are similar degrees of variations in the modeling of wettability of hBN. Over the past decade, molecular dynamics (MD) simulations have also been conducted in studying the interfacial properties of hBN^27^ and the calculated WCA are shown in Figure 1. In one of the early studies, Li and Zeng employed quantum molecular dynamics (QMD) simulations of water nanodroplets on a 1L hBN sheet and yielded a contact angle of 86°.^23^ Subsequently, Tocci et al. employed ab initio molecular dynamics (AIMD) and obtained a comparable value of 87° for the contact angle of 1L hBN.^24^ In a recent research by Govind Rajan et al., the contact angle of water on the bulk hBN basal plane was found to be 81°.^1^ Additionally, Kumar Verma and Govind Rajan observed that surface roughness significantly influences the WCA, and 1L hBN exhibited a hydrophobic nature with a contact angle of 90° in the presence of extremely low surface density of vacancy defect and exposed-edge.^28^ Most recently, Luo et al. developed a polarizable force field grounded in quantum chemical simulations to accurately model the complex interactions and polarization effects at the hBN-water interface. This new theoretical framework, incorporating the anisotropic polarizability of hBN and partial atomic charges, allowed for the self-consistent calculation of water-induced polarization and hBN-water binding energies. This MD simulation work predicted a WCA value on bulk hBN around 83°.^29^ However, several other MD simulation studies reported WCA ranging from 55 to 73° for different hBN samples, indicating the challenges in uncovering the water wettability of hBN.^16,21,22,25−27^

The controversy on the water wettability of hBN from both experimental and simulation studies shown in Figure 1 could result from the following two factors: airborne hydrocarbon contaminants and defects and quality of the hBN samples. The adsorption of airborne hydrocarbons is difficult to avoid and it masks the intrinsic wettability and therefore has a significant impact on the experimentally measured WCA.^31,32^ A notable illustration is the considerable change in the WCA of freshly synthesized graphene on copper via CVD (e.g., WCA changes from 42 to 90°),^32^ following the adsorption of airborne hydrocarbons. Environmental contaminants affect not only graphene but also other 2D materials like hBN, MoS_2_, and mica.^5,19,30,32^ In a similar vein, chemical defects often increase the hydrophilicity of materials due to their polar nature. Kozbial et al. previously proposed that static WCA may inadequately capture the intrinsic wettability of pristine graphite due to the sample defect. They observed that while the static and receding WCA might decrease with the increase of the defect density, the advancing WCA is almost independent of the defect density, rendering it a more reliable parameter characterizing the intrinsic wettability.^33^ Given that pristine graphite and hBN crystals exhibit analogous structural and physical similarities, the initial hypothesis and methodology employing the dynamic WCA technique for characterizing pristine graphite may also be suitably extended to assess the intrinsic wettability of hBN.

The aforementioned disparities in the WCA of hBN could potentially be attributed to surface defects arising from the utilization of hBN samples of various qualities. Indeed, the quality of hBN crystals is not as good as that of graphite. Numerous techniques, for the preparation of graphene, have been used to date to synthesize hBN nanosheets,^12,13,20^ including exfoliation methods from bulk materials (e.g., mechanical, chemical, thermal, etc.), intercalation approaches, solid and liquid phase sonification methods, mechanical milling techniques, chemical solution derived (CSD) techniques, CVD, and PLD.^5−7,20,31,34,35^ It is important to note that the synthesis of high-quality hBN is still very challenging and the quality of the resulting nanosheets is yet to be improved. The bulk hBN crystals produced thus far have exhibited lower quality and smaller dimensions in comparison to those of highly ordered pyrolytic graphite (HOPG). Meanwhile, there has not been any dynamic WCA measurement reported to elucidate the effect of the defects, which could be the key to explain the previous inconsistency and uncover the intrinsic WCA of hBN.

In the present study, using the highest quality (lowest surface roughness) hBN commercially available, for the first time, we studied the effect of airborne hydrocarbon and surface defect on water wettability of hBN. We experimentally measured dynamic contact angles, i.e., advancing and receding contact angles, beyond commonly studied static WCA on both freshly mechanically exfoliated and aged hBN. Our results suggest that the static WCA of hBN (64 ± 5°) does not represent the intrinsic water wettability of freshly exfoliated hBN due to the existence of defects. Instead, the advancing WCA (79 ± 3°) is a more precise indicator of the intrinsic water wettability, similar to that previously observed in pristine graphite.^33^ Our results also showed that airborne hydrocarbons increase the WCA of hBN as expected. Based on the experimental data, a model has been proposed to describe the effect of airborne hydrocarbon contaminants and defects on both static and dynamic WCA.

## Experimental Section

In this study, all hBN samples (HQ graphene, The Netherlands)^19^ were prepared by mechanical exfoliation using scotch tape. For the freshly exfoliated samples, WCA measurements were promptly conducted within two min after exfoliation to minimize airborne contamination. The aged hBN samples were exfoliated and stored inside glass Petri dishes in an ambient atmosphere for a minimum of 1 month.

The static and receding WCA values were measured using the VCA Optima XE contact angle tester (AST Products, Inc.). The static WCA of freshly exfoliated hBN was determined using the sessile drop method. Due to the very small sample size (0.5 × 0.5 mm), a DI water droplet with a volume less than 0.5 μL was carefully deposited onto the freshly exfoliated hBN surface. The static WCA was immediately measured after droplet deposition, and at least three different samples were examined to ensure reproducibility. For the receding WCA, the evaporation method was employed by depositing a water droplet with a volume less than 0.5 μL onto the freshly exfoliated hBN surface and allowed to evaporate at rt, enabling water to recede from the wetted region. Furthermore, the advancing WCA measurements were carried out utilizing an Attension Theta Lite Tensiometer (Biolin Scientific, Sweden) with a 0.5 μL syringe (Hamilton Company). For the advancing WCA measurement, a DI water droplet with a volume of less than 0.2 μL was initially deposited on the hBN surface. Subsequent addition of water was made to the droplet, and the advancing WCA was determined when the three-phase contact line jumped outward.

Atomic force microscopy (AFM) imaging of both freshly exfoliated and aged hBN samples was performed in tapping mode by using a Bruker AFM system. The resulting AFM images were analyzed by using NanoScope Analysis software. X-ray photoelectron spectroscopy (XPS) was employed to study the chemical composition of freshly exfoliated and aged hBN samples using a Thermo Fisher ESCALAB 250 Xi instrument. To minimize potential airborne contamination, bulk hBN crystals were mechanically exfoliated within the XPS vacuum chamber using a similar method previously reported for HOPG.^36^ The mechanical exfoliation process involved affixing Scotch tape to both the top surface of an hBN crystal and the transfer rod within the preparation chamber. Subsequently, the preparation chamber was evacuated to a pressure of 4.3 × 10^–7^ Pa. The prepared sample was then transferred to a sample holder inside the analysis chamber. Upon withdrawal of the transfer rod from the analysis chamber, the motion induced cleavage of the hBN crystal, resulting in a freshly exfoliated hBN sample within the analysis chamber. Subsequent XPS scans were performed inside the analysis chamber at 5.7 × 10^–9^ Pa. Aged hBN samples were directly transferred to a sample holder inside the analysis chamber for XPS scans. The obtained XPS spectra were analyzed by using CasaXPS software.

## Results and Discussion

### XPS of Freshly Exfoliated and Aged hBN

**Figure 2:**
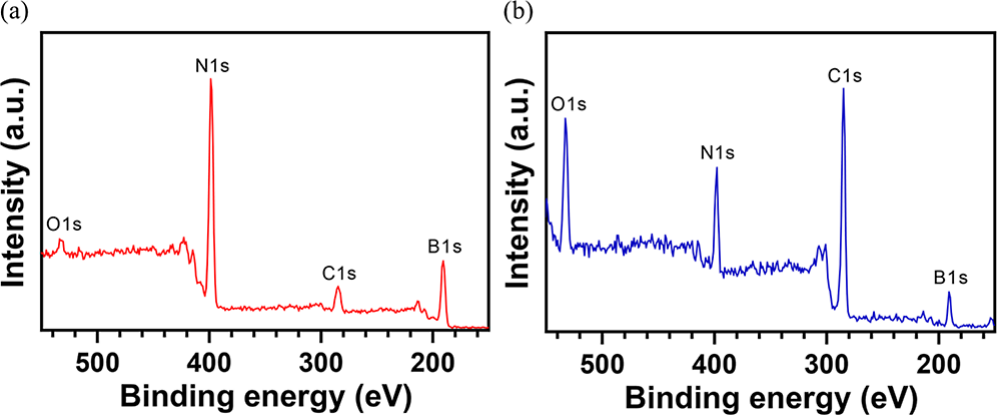
XPS survey scans: (a) freshly mechanically exfoliated hBN within XPS analysis chamber and (b) aged hBN directly transferred to analysis chamber.

XPS was conducted on both freshly exfoliated and aged hBN samples, and the results are presented in Figure 2. The XPS survey scan for freshly exfoliated hBN (Figure 2a) exhibited characteristic peaks for boron and nitrogen at approximately 190 and 399 eV, respectively, aligning well with established literature values.^37−42^ The nearly 1:1 ratio of boron to nitrogen atomic percentage observed in the survey scan reaffirms the stoichiometry of hBN (Table 1). Minor peaks corresponding to carbon and oxygen, at approximately 10 and 2% atomic percentage, respectively, indicate relatively low levels of airborne contamination on the freshly exfoliated hBN. Despite the exfoliation process occurring within the XPS vacuum chamber, it is challenging to entirely avoid airborne contamination, which might arise from scotch tape or residue pump oil vapor in the vacuum chamber. In contrast, the XPS survey scan for the aged hBN (>1 month in ambient) exhibited a significant increase in both carbon and oxygen peaks, reaching approximately 59 and 12% atomic percentage, respectively, indicating a substantial deposition of airborne contaminants on the aged hBN surface (Figure 2b).^31^ Furthermore, a 9% increase in the boron-to-nitrogen atomic ratio (from 1.08 to 1.18) suggests a potential diminishment of nitrogen content attributed to oxidation, given that oxygen has a higher affinity to occupy atomic positions over nitrogen as opposed to boron.^43^

**Table 1:** XPS Survey Spectrum Atomic Percentage of Selected Elements

element	position (eV)	atomic percentage	
		freshly exfoliated hBN (%)	well-aged hBN (%)
B 1s	191	45.70	15.42
C 1s	285	10.07	59.30
N 1s	398	42.20	13.11
O 1s	533	2.02	12.16

### AFM of Freshly Exfoliated and Aged hBN

**Figure 3:**
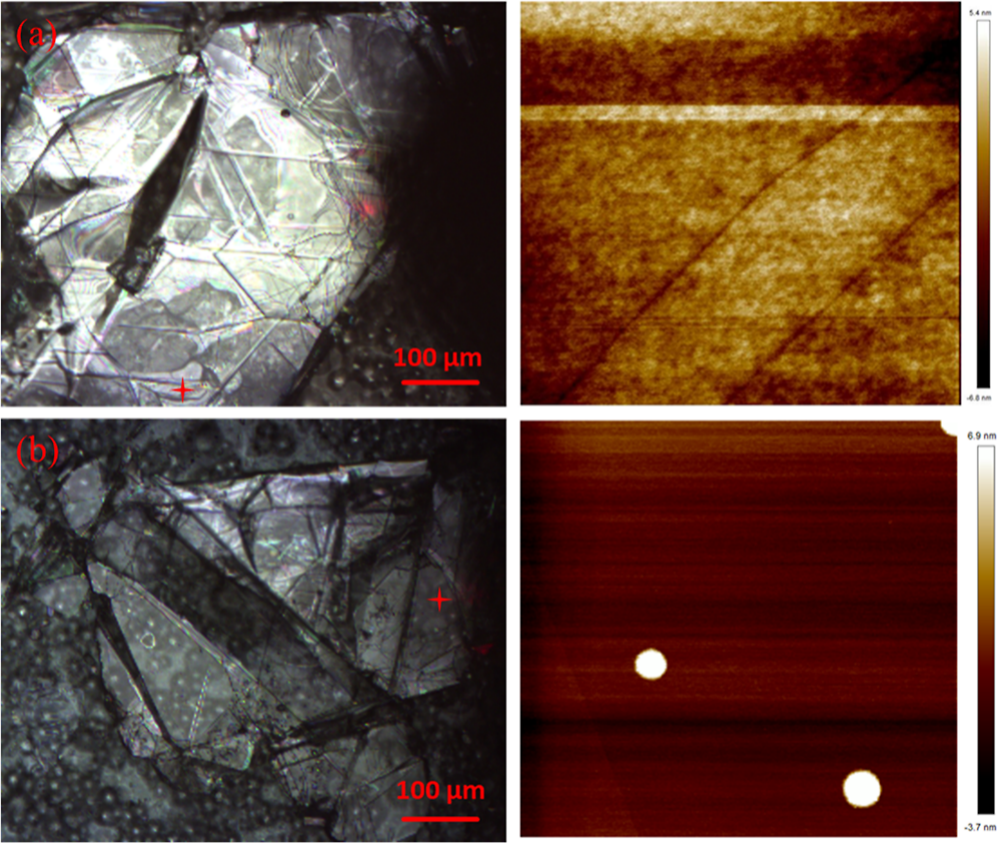
Optical and AFM images of (a) freshly exfoliated hBN and (b) aged hBN. Optical images (left) are taken at 5× magnification with a 100 μm scale bar. Red cross symbols on optical images indicate the location of AFM scans. AFM images (right) scan area of (a) 10 × 10 μm and (b) 5 × 5 μm.

Figure 3 presents the optical and AFM images of both freshly exfoliated and aged hBN samples. The optical images showed a notable abundance of defects on the micrometer scale, including line defects, step edges, and fractures. AFM scans were meticulously performed near the vicinity of the contact line between the wetted and unwetted regions of the crystal (see discussions of WCA below). The quantitative assessment of surface roughness (Ra) yielded a measurement of 1.7 nm for the freshly exfoliated specimen and 1.4 nm for the aged counterpart. Upon juxtaposition, it was ascertained that significant defects persisted in both samples, while the surface unevenness in regions devoid of defects demonstrated similar values for both samples. Drawing a comparison with graphite, the surface roughness of areas with few defects closely resembled that of high quality HOPG from SPI, approximating 1.4 nm. Nevertheless, the surface roughness for top-tier HOPG (Momentive) specimens can descend to remarkable values, as low as 0.353 nm, in contrast to inferior quality pyrolytic graphite, which can exhibit values as elevated as 30 nm.^33^ Notably, the AFM image of the aged sample shows micrometer-sized particles, presumably dust particles, on the flat surface (Figure 3b). Considering the substantial aging period (>1 month), it is plausible that airborne contaminants have fully covered the substrate without adversely affecting the original large defects.

### Static and Dynamic WCA on Freshly Exfoliated and Aged hBN

**Figure 4:**
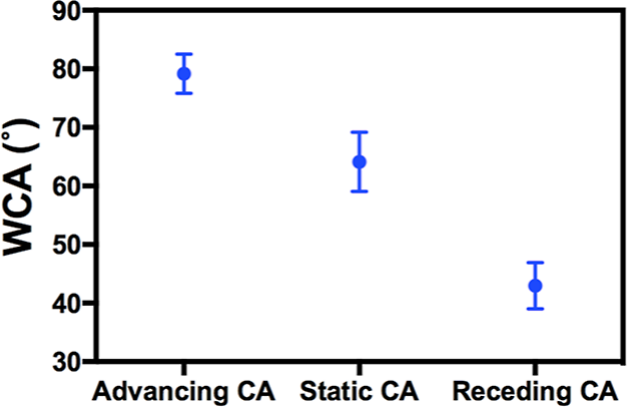
Static and dynamic WCA on freshly exfoliated hBN. Crystal size: ∼ 1 × 1 mm. Water droplet size <0.5 μL. At least three sample replicates.

Figure 4 presents the static and dynamic WCA of freshly exfoliated hBN samples. The static WCA value was found to be 64 ± 5°, which is consistent with some previous reports.^5,17,20^ The advancing WCA was found to be 79 ± 3°, while the receding WCA was found to be 43 ± 4°. It is worth noting that no prior research reported experimental values of advancing and receding WCAs of freshly exfoliated hBN, likely due to the very small size and hBN crystal available.^6^

**Figure 5:**
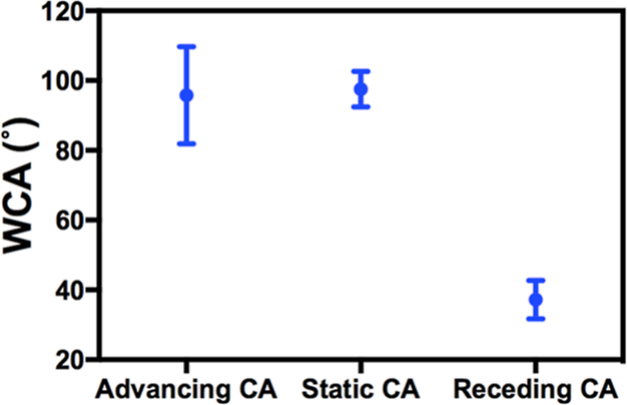
Static and dynamic WCA on aged (>1 month) hBN. Crystal size: ∼ 1 × 1 mm. Water droplet size <0.5 μL. At least three sample replicates per category.

Static and dynamic WCA measurements on aged hBN samples are conducted as well. As shown in Figure 5, the static WCA was found to be 98 ± 5° on the aged hBN samples. Meanwhile, the advancing and receding WCAs were measured to be 96 ± 13° and 37 ± 5°, respectively. The hydrophobic behavior exhibited by the static WCA is attributed to the coverage of the substrate by airborne contaminants.^32^ However, the receding WCA value is very close to that of freshly exfoliated hBN.

Previously, Kozbial et al.^33^ proposed a mechanism to explain why the advancing WCA, instead of the static WCA, represents the intrinsic wettability of freshly exfoliated pristine graphite. They found that both static and receding WCA was influenced by defects, and the WCA values reflect the wettability of a composite surface consisting of both defect-laden and pristine graphite regions. They concluded that the advancing WCA reflects the intrinsic water wettability of pristine graphite.^33^

The atomic-scale surface morphology of hBN is characterized by a hexagonal lattice architecture comprising alternately positioned boron and nitrogen atoms. Within this configuration, each boron atom is tricoordinated with nitrogen atoms, and reciprocally, each nitrogen atom is tricoordinated with boron atoms. This atomic arrangement establishes a stoichiometric ratio of 1:1 between boron and nitrogen, imparting chemical uniformity to the surface while simultaneously inducing electronic polarization. In the context of MD simulations, Luo et al. have previously elucidated that within the proximal hydration layer a predominant orientation of water molecules is tangential relative to the hBN surface. This orientation contrasts with the behavior of water molecules situated beyond the initial hydration layer, which display no discernible preferential alignment.^29^ Pertaining to the investigation of defects, which are on the order of micrometers, a stochastic distribution of defects was depicted schematically, highlighting the scale and nature of imperfections within the hBN structure.

**Figure 6:**
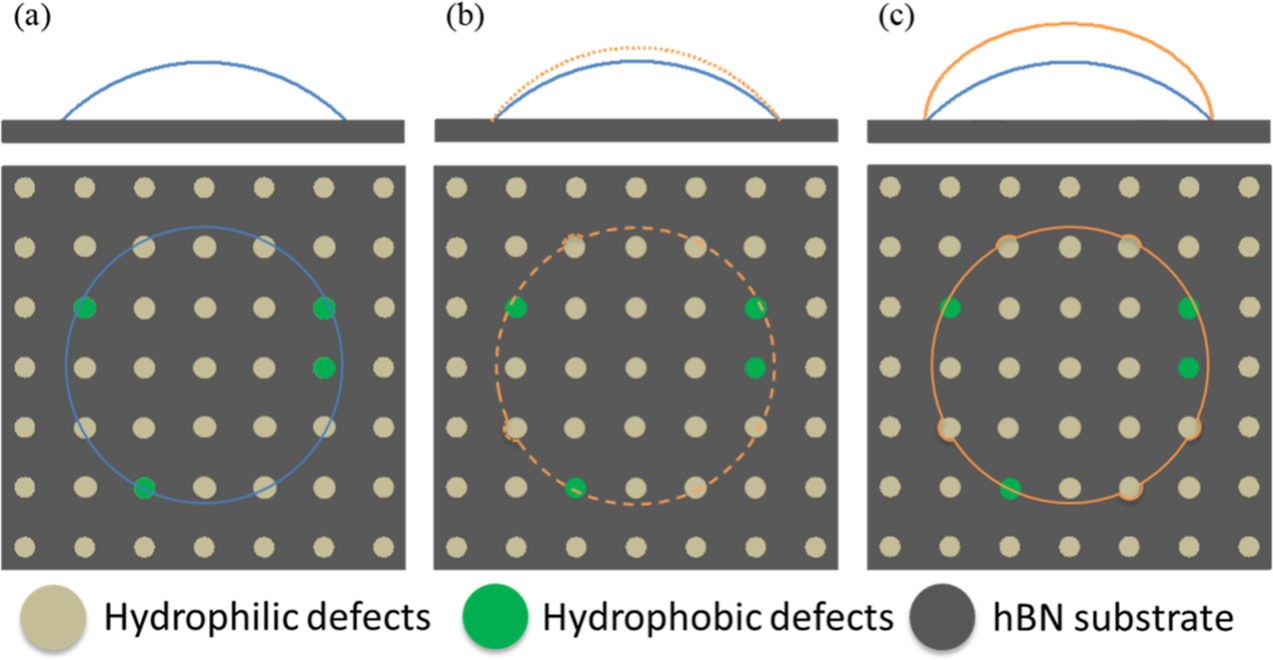
Effect of defect and hydrocarbon contaminant on the advancing WCA. Side view in the upper figures and top view in the lower figures. The contact line is in blue and orange solid/dash lines. Background dark gray area represents the pristine hBN surface. Brown and yellow solid circles represent hydrophilic defects and airborne hydrocarbons, respectively. (a) The contact line interacts with hBN, hydrophilic defects, and airborne hydrocarbons when a water droplet is placed on the sample. (b) The contact line pinned by the relatively hydrophobic hBN and airborne hydrocarbons while water begins to advance onto (wet) the hydrophilic defects, generally increasing WCA. (c) The contact line completely wets the hydrophilic defects and is still pinned by h-BN and airborne hydrocarbons. Further addition of water into the droplet results in the outward jump of the contact line, which corresponds to the advancing WCA. Note: schematics are not to scale (the drawing is partially adapted with permission from ref (33). Copyright 2017 American Chemical Society.).

Based on the theoretical framework of dynamic WCA on pristine graphite and the experimental data presented in this study, here, we propose a modified qualitative model elucidating the influence of both airborne hydrocarbons and surface defects on the static and dynamic WCA of hBN. As depicted in Figure 6a, when a water droplet is placed on the hBN surface, the 3-phase contact line interacts with hBN, as well as more hydrophobic airborne hydrocarbon contaminants (represented by yellow circles) and more hydrophilic surface defects (e.g., line defects and step edges, represented by brown circles). In this initial state, without adding or withdrawing water from the droplet, the figure illustrates the static WCA. When additional water is introduced into the droplet (as depicted in Figure 6b), the contact line selectively wets the more hydrophilic surface defects. Meanwhile, the contact line remains unaffected on the airborne hydrocarbons and hBN. The model depicted in Figure 6c encapsulates the final state before the contact line jumps outward macroscopically, characterized by its complete wetting on the hydrophilic defect. These schematics qualitatively describe the localized movement of the water contact line until the WCA reaches its maximum value, i.e., the advancing WCA. In accordance with this model, the advancing contact angle is primarily dictated by the intrinsic wettability properties of hBN, if there are minimal airborne hydrocarbons (i.e., freshly exfoliated hBN). For aged hBN extensively covered with airborne hydrocarbons, the advancing contact angle is determined by both hBN and airborne hydrocarbons. Notably, in this scenario, the presence of hydrophilic defects does not affect the advancing contact angle.

**Figure 7:**
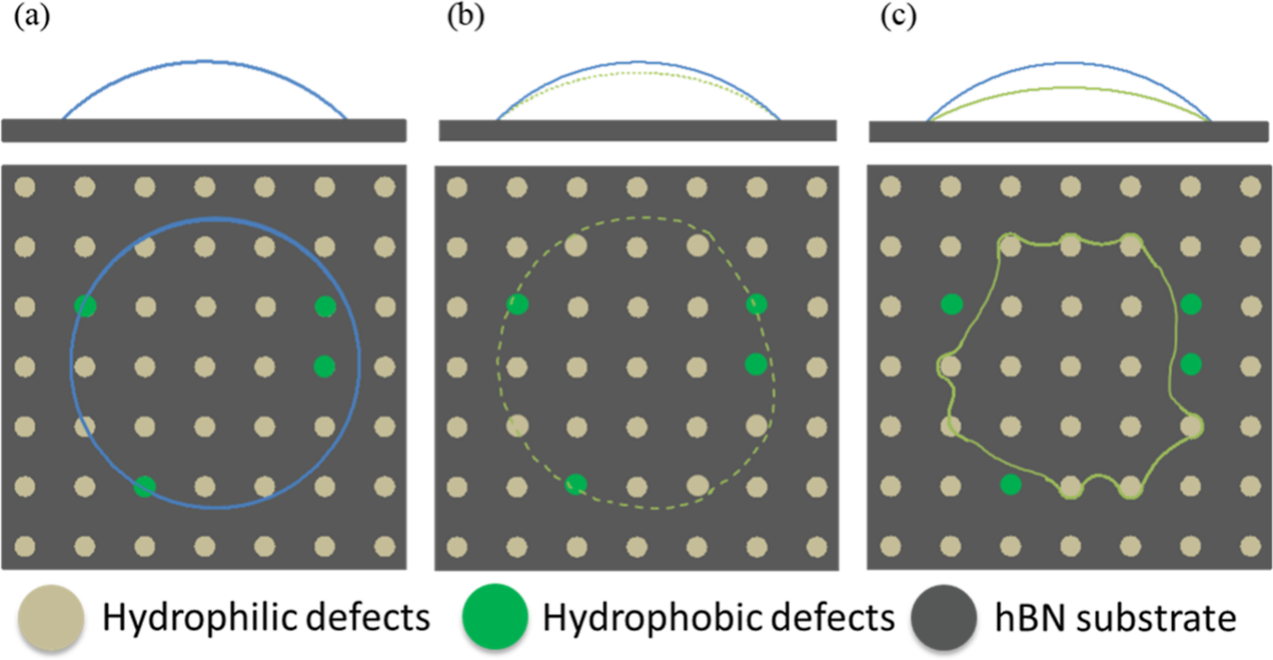
Effect of defect and hydrocarbon contaminant on the receding WCA. Side view in the upper figures and top view in the lower figures. The water contact line is in blue and green solid/dash lines. Background dark gray area represents the substrate surface. Brown and yellow solid circles represent hydrophilic defects and airborne hydrocarbons, respectively. (a) Contact line interacts with hBN, hydrophilic defects, and airborne hydrocarbons when a water droplet is placed on the sample. (b) The contact line becomes pinned on hydrophilic defects while the drop begins to recede (dewet) in the most hydrophobic area (i.e., airborne hydrocarbons) as the liquid is withdrawn, generally decreases WCA. (c) The contact line is pinned by hydrophilic defects and completely dewets from the airborne hydrocarbons. Further withdrawal of water from the droplet will result in an inward jump of the contact line, which corresponds to the receding WCA. Note: schematics not to scale (the drawing is partially adapted with permission from ref (33). Copyright 2017 American Chemical Society.).

Regarding the receding contact angle, the initial state (i.e., static WCA) is the same as the advancing WCA (Figure 7a). As water is extracted from the droplet (as illustrated in Figure 7b), while the water contact line does not dewet from the more hydrophilic surface defects, it selectively dewets from the most hydrophobic airborne hydrocarbons. As shown in Figure 7c, in the final state before the contact line jumps inward macroscopically, which corresponds to the receding WCA, the contact line completely dewets from the airborne hydrocarbons. In other words, airborne hydrocarbons do not impact the receding contact angle which is only determined by the hydrophilic surface defects and the intrinsic hBN wettability.

According to this model, the static WCA measurements are determined by not only the intrinsic wettability of the hBN but also airborne hydrocarbons and hydrophilic defects, as illustrated in Figures 6a and 7a. Consequently, static WCA does not accurately represent the intrinsic wettability of hBN. The advancing WCA is larger than the static WCA and is determined by the most hydrophobic component of the substrate, intrinsic hBN and/or the hydrophobic hydrocarbon contaminants and not influenced by the hydrophilic defects (Figure 6c). Conversely, the receding WCA is determined by the most hydrophilic components of the substrate, i.e., defect, and is lower than the static WCA.

Our experimental data are consistent with the proposed model. In the absence of airborne contaminants, as in the case of freshly exfoliated hBN, advancing WCA reflects the intrinsic wettability of hBN. The static WCA is between the advancing and receding WCAs, indicating influence from both the intrinsic wettability and defects. For aged hBN samples, where there is a significant amount of airborne hydrocarbons on the surface, both static and advancing WCAs are determined by not only the intrinsic wettability of hBN but also the airborne hydrocarbons, resulting in higher values compared to freshly exfoliated hBN. Since the sample is well-aged, airborne hydrocarbon contaminants dominate over the intrinsic wettability of hBN, resulting in similar advancing and static WCA values. Conversely, the receding WCA is not impacted by the airborne hydrocarbons, as depicted in Figure 7c. As a result, the receding WCAs of freshly exfoliated and aged hBN are almost the same, as shown in Figures 4 and 5.

## Conclusions

In summary, to uncover the intrinsic water wettability of hBN, we investigated the effect of airborne hydrocarbons and intrinsic defects on both static and dynamic WCAs. XPS results confirmed the adsorption of airborne hydrocarbons after aging hBN samples in ambient. AFM results indicated the existence of various defects for both freshly exfoliated and aged hBN samples. Our WCA results showed that both the receding and the static WCA are impacted by defects, which suggests that previously reported static WCAs do not characterize the intrinsic water wettability of hBN. Instead, we found that the advancing WCA of freshly exfoliated hBN is not impacted by the defects and airborne hydrocarbons. As a result, the advancing WCA on freshly exfoliated hBN, determined to be 79° ± 3, best represents the intrinsic water wettability of hBN. Nevertheless, the advancing WCA as a metric for intrinsic wettability may be applicable predominantly to graphite and hBN. Conversely, for other 2D materials such as MoS_2_, an alternative metric might be more representative, wherein a receding WCA could serve as an indicator of intrinsic wettability.^30^ To broaden the applicability, the qualitative framework proposed may extend to a wider array of 2D materials, thereby facilitating a deeper comprehension of their inherent wettability. A qualitative model has been proposed to describe the effect of airborne hydrocarbons and defects on the static and dynamic WCA of hBN, which is consistent with the experimental results.

## Abbreviations

AFM: atomic force microscopy
AIMD: ab initio molecular dynamics
BN: boron nitride
CSD: chemical solution derived
CVD: chemical vapor deposition
hBN: hexagonal boron nitride
HOPG: highly ordered pyrolytic graphite
MD: molecular dynamics
ML: multilayer
PLD: pulsed laser deposition
QMD: quantum molecular dynamics
vdW: van der Waals
WCA: water contact angle
XPS: X-ray photoelectron spectroscopy
1L: monolayer
2D: two-dimensional
